# A review of the neural control of micturition in dogs and cats: neuroanatomy, neurophysiology and neuroplasticity

**DOI:** 10.1007/s11259-022-09966-9

**Published:** 2022-07-08

**Authors:** Floriana Gernone, Annamaria Uva, Arianna Maiolini, Andrea Zatelli

**Affiliations:** 1 Department of Veterinary Medicine, University of Bari, Bari, Italy; 2 Vetsuisse Fakultat - Universitat Bern, Bern, Switzerland

**Keywords:** Urination, Micturition, Neurogenic bladder, Canine, Feline, Humans

## Abstract

This article discusses the current knowledge on the role of the neurological structures, especially the cerebellum and the hypothalamus, and compares the information with human medicine. Micturition is a complex voluntary and involuntarily mechanism. Its physiological completion strictly depends on the hierarchical organisation of the central nervous system pathways in the peripheral nervous system. Although the role of the peripheral nervous system and subcortical areas, such as brainstem centres, are well established in veterinary medicine, the role of the cerebellum and hypothalamus have been poorly investigated and understood. Lower urinary tract dysfunction is often associated with neurological diseases that cause neurogenic bladder (NB). The neuroplasticity of the nervous system in the developmental changes of the mechanism of micturition during the prenatal and postnatal periods is also analysed.

## Introduction

Micturition is a two-stage storage process and periodically voiding urine (Labato and Acierno 2010). The term micturition is etymologically more appropriate than urination as the latter refers exclusively to urine evacuation. Micturition requires physiological competence and the proper functioning of both the urinary bladder and the urethra and depends on the behaviour that develops during nervous system maturation and individual experience. The physiological competence between the urinary bladder and urethra results from the compliance of the central nervous system (CNS) and peripheral nervous system (PNS), including sympathetic and parasympathetic components. Consequently, disease of the CNS or the peripheral or autonomous system may lead to neurogenic bladder (NB) dysfunction. NB refers to the lower urinary tract disease (LUTD) caused by neurological disease (Lane 1995), and the site and the origin of the neurological lesion influence the pattern of dysfunction. Disorders of urine storage usually lead to urinary incontinence: affected animals leave a pool of urine where they have been lying or may dribble urine while walking. The coat around the vulva or prepuce may be wet, and perivulvar or peripreputial dermatitis can result from urine scalding.

On the other hand, alteration in urine voiding leads to urinary retention (Labato and Acierno 2010). Failure of normal voiding is characterised by frequent attempts to urinate with stranguria and the passage of only small amounts of urine. Animals with abnormalities of the voiding phase may develop overflow incontinence due to the dribbling of urine associated with bladder overdistention, which causes detrusor muscle fibres failure (tight-junctions). This failure can irreversibly compromise the future contractile function of the detrusor muscle. In human medicine, LUTD has an enormous impact on the quality of life (QoL) in affected patients because of the physical and psychosocial consequences (Panicker 2020). In dogs and cats as well, urinary (and faecal) control is essential for owner management and, as in humans, represents a severe concern regarding the increased risk of urinary tract infections (UTIs) that impact QoL (Freeman et al. 2013; Dinh et al.^2019^).

For these reasons, the clinician needs to localise the neurological lesion responsible for the urinary incompetence and suggests the best way to manage the condition and choose the appropriate treatment. Detailed information on neurophysiology is also provided, including for human medicine and focus on the cerebellum and hypothalamus pathways involved in micturition.

The literature reviewed was based on MEDLINE/Pubmed (http://www.ncbi.nlm.nih.gov/pubmed/) and the Web of Science (http://www.webofscience.com). The keywords used were “micturition”, “urination”, “neural control”, “neurophysiology”, “bladder dysfunction”, “neurogenic bladder”, “dog”, “canine”, “cat”, “feline”, and “humans”. The search of PubMed was conducted using the Boolean Operators AND and OR [micturition” OR “urination”, OR “neural control”, OR “neurophysiology”, OR “bladder dysfunction”, OR “neurogenic bladder AND (dog OR canine) (cat OR feline) humans]. In addition, the Textbook of Veterinary Internal Medicine by Ettinger et al., 8th ed., and veterinary neurology books (De Lahunta et al., Veterinary Neuroanatomy and Clinical Neurology,4^th^ ed, Lorenz et al., Handbook of Veterinary Neurology, 4^th^ ed.) were also reviewed.

### Neuroanatomy

#### Lower motor neurons: storage and voiding reflex (Fig.1a,1b)

**Fig. 1:**
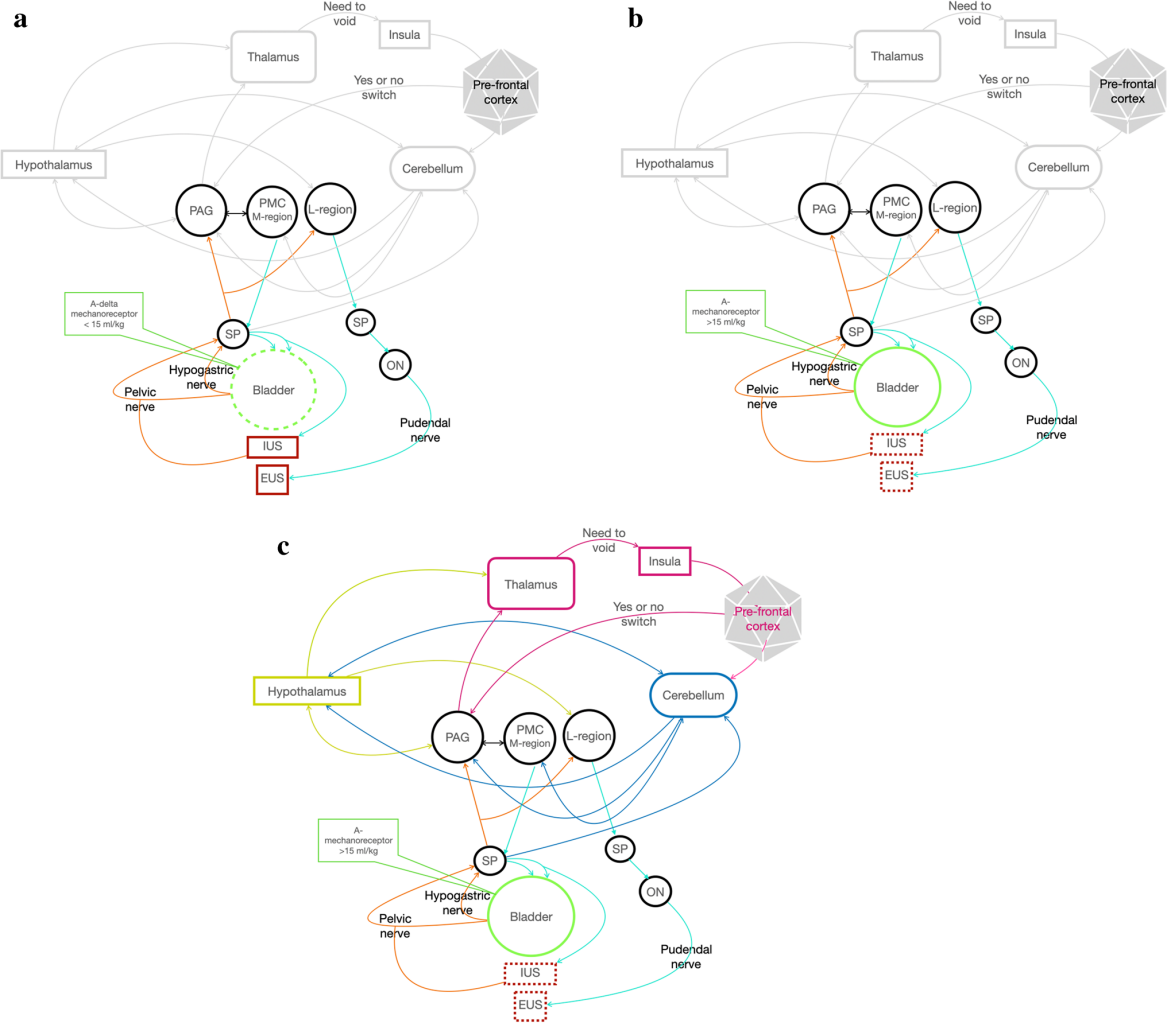
**a**, **b**, and **c** storage, voiding, micturition. EUS: external urethral sphincter; IUS: internal urethral sphincter; SP: sacral parasympathetic; ON: Onuf’s nucleus; PMC: pontine micturition centre; PAG: periaqueductal grey nucleus; orange line: sensory information travelling along the pelvic nerve and hypogastric nerve and Spinobulbar tract. Turquoise line: afferent tract (reticulospinal tract). a Storage phase. Green dash line: relaxation. Red line: contraction. During the storage phase, A-delta-mechanoreceptors record bladder stretching, and the impulse travels along the hypogastric nerve and pelvic nerve. The efferent impulses run across the spinobulbar tract and reach PAG. PAG inhibits PMC and, through the reticulospinal tract, if the bladder isn’t entirely filled, the impulse reaches neuronal cells body of the hypogastric nerve, pelvic nerve and pudendal nerve to prevent urine leakage and guarantee continuing urine filling. In this manner, the bladder is relaxed while IUS and EUS continue to be contracted to avoid urine leakage. b Voiding phase. Green line: contraction. Red dash line: relaxation. During the voiding phase, A-delta mechanoreceptors register a stretch more significant than 15 ml/kg, and the efferent impulse travels along the spinobulbar tract reaching PAG. PAG excites PMC and L-region, running across the reticulospinal tract, through hypogastric and pelvic nerves inducing bladder contraction and IUS relation. Contemporary brainstem L-region sends information through the bulbospinal tract to the pudendal nerve through ON for EUS relaxation. c Micturition. Green line: contraction. Red dash line: relaxation. The urine voiding reflex is under the highest centre control (thalamus, insular and pre-frontal cortex) integrated by the hypothalamus and cerebellum. When PAG receives information about the fullness of the bladder, it sends information to the thalamus, insula and pre-frontal cortex. The integration with the pre-frontal cortex allows deciding if voiding (switch or not), depending on an appropriate site, learned behaviours. On the contrary, the pre-frontal cortex inhibits the switching, postponing the timing for voiding. The information is also integrated with the hypothalamus for meeting the needs to mark the territory, for example. The cerebellum receives information from pelvic and pudendal nerves, integrates information between the pre frontal cortex, hypothalamus and PAG and, bidirectionally, with PMC. Cerebellum modulates and coordinates micturition

Anatomically, the LUT consists of the urinary bladder, urethra, internal urethral sphincter (IUS) and external urethral sphincter (EUS), and normal function of these organs and tissues is required for an appropriate micturition. The body of the urinary bladder is composed of three layers of smooth muscle, which make up the detrusor muscle (Levin et al. 1994). Muscle bundles in the bladder are organised in circles, with extensive collagen and elastin supporting structure to promote distensibility (Purinton and Oliver Jr 1979). To respond to the nervous and hormonal control systems, each part of the urinary tract muscles need specific receptors for the transmitters/modulators, which are released from nerves or generated locally, and the associated cellular pathways for initiating contraction and relaxation (Levin et al. 1994). The detrusor muscle contains adrenergic and cholinergic (muscarinic) receptors (Labato and Acierno 2010; Levin et al. 1994). Sensory receptors (stretch and pain) are also present within the detrusor muscle wall (Oliver et al. 1969; Purinton and Oliver Jr 1979; Levin et al. 1994; Labato and Acierno 2010; Uemura 2015; De Lahunta et al. 2015). Smooth muscle in the detrusor muscle contributes to form the IUS, a contiguous system extending down to the urethra with a length of up to two inches in cats and half an inch in humans (Griffiths 1891). The detrusor muscle and IUS contain *alfa-*adrenergic receptors (Oliver et al. 1969; Purinton and Oliver Jr 1979; Labato and Acierno 2010; Levin et al. 1994; Uemura 2015; De Lahunta et al. 2015). The EUS is formed by striated muscle fibres encircling the distal urethra and contains nicotinic receptors (Oliver et al. 1969; Purinton and Oliver Jr 1979; Levin et al. 1994; Labato and Acierno 2010; Uemura 2015; De Lahunta et al. 2015). Pain and stretch receptors are also present within the wall of the urethra.

The urinary bladder has two critical functions: storing urine for an extended time without leakage and rapid emptying. Storage of urine occurs at low pressure, meaning the bladder relaxes during the filling phase. Disturbances in the storage function may result in LUT symptoms, such as urgency, frequency, and urge incontinence (Labato and Acierno 2010). Emptying requires the coordinated contraction of the bladder and relaxation of the urethra. Disturbances in the voiding function can lead to urinary retention (Labato and Acierno 2010). From a functional point of view, storage and periodically voiding of urine involve a complex interaction between the somatic and autonomic nervous systems, coordinated by supraspinal pathways involving the brainstem, cerebral cortex and cerebellum, establishing the interaction between voluntary and involuntary control. The impulses travel along with three pairs of nerves, thoracolumbar sympathetic nerves (hypogastric nerves and sympathetic chain), sacral parasympathetic (pelvic nerves) and sacral somatic nerves (pudendal nerves) (Oliver et al. 1969; Purinton and Oliver Jr 1979; Uemura 2015; De Lahunta et al. 2015). The hypogastric nerve origins from the lumbar spinal nerves (L1-L4 in dogs, L2-L5 in cats) (Oliver et al. 1969; Purinton and Oliver Jr 1979; Uemura 2015; De Lahunta et al. 2015). The pre-ganglion sympathetic (General Visceral Efferent axons, GVE) neuronal cell bodies are located in the lateral grey column in the L1-L4 and L2-L5 spinal segments in dogs and cats, respectively (Oliver et al. 1969; Purinton and Oliver Jr 1979; Uemura 2015; De Lahunta et al. 2015). The pre-ganglion sympathetic axon synapses in the caudal mesenteric ganglion, together with the post-ganglion sympathetic axons, which, together with the pelvic nerve, form the pelvic plexus (De Lahunta et al. 2015). At the pelvic plexus, one group of post-ganglion sympathetic axons (alfa-adrenergic) synapses with alfa-mechanoreceptors on the bladder neck and IUS. Another group of sympathetic post-ganglion axons (beta-adrenergic) synapses on beta-receptors of the entire wall bladder, inhibiting the muscle contraction and allowing further expansion of the bladder wall for urine storage (De Lahunta et al. 2015). The pelvic nerve origins from the first, the second and the third sacral nerves and reaches the urinary bladder (Uemura 2015; De Lahunta et al. 2015). The pelvic nerve comprises GVE parasympathetic axons and general visceral afferent (GVA) axons, and the latter detect the stretching of the bladder wall. The pre-ganglion parasympathetic neuronal cell bodies are located in the lateral grey column of the sacral spinal cord segments (S2-S3) (Uemura 2015; De Lahunta et al. 2015). The post-ganglion parasympathetic axons, synapsed on the pelvic plexus, innervate the muscarinic cholinergic receptors in the detrusor muscle (Uemura 2015; De Lahunta et al. 2015). The pudendal nerve arises from the ventral branches of all three sacral nerves (Uemura 2015; De Lahunta et al. 2015). It has a general somatic efferent (GSE) function, and the neuronal cell bodies, i.e.“Onuf’s nucleus” described by neuropathologist Onufrowicz more than a century ago (Onuf-Onufrowicz 1899), are located in the ventral grey column of the sacral segments (Uemura 2015; De Lahunta et al. 2015). Axons from these motor neurons, through the pudendal nerve, excite the EUS muscle via cholinergic receptors supporting sphincteric contraction and promoting continence (Fowler et al. 2008). There are considerable variations among different species regarding the Onuf’s nuclei location in ventral spinal cord grey matter (Sato et al. 1978; Kuzuhara et al. 1980; Roppolo et al. 1985).

The axons travel through the ventral roots, enter the spinal nerves, continue in their ventral branches and contribute to the sacral plexus in the pelvic cavity (De Lahunta et al. 2015). The pudendal nerve leaves the sacral plexus to innervate the nicotinic receptors, thus providing the voluntary contraction of this muscle (De Lahunta et al. 2015). The pudendal nerve also carries sensory information (GSE) from the wall of the urethra, detecting their stretching, urine flow and pain (De Lahunta et al. 2015).

All the afferent components of these three nerves are composed of two kinds of axons: Aδ and C fibres, myelinated and unmyelinated, respectively (De Groat 1995, Blok and Holstege 1998, De Groat and Wickens 2013). The Aδ fibres are located in the detrusor smooth muscle layer and respond to passive distension and active contraction. C-fibres are more widespread and are insensitive to bladder filling in physiological conditions. Many studies in cats and rats confirm that C-fibres respond to noxious stimuli and are activated after suprasacral spinal cord injury (SCI) (De Groat 1995; Blok and Holstege 1998; De Groat and Wickens 2013).

Physiologically, all this information travels up and down these nerves, whose work is integrated by the supraspinal centre, enabling coordinated urine storage and voiding (De Lahunta et al. 2015).

#### Brainstem integration: Pontine Micturition Centre, L-region nucleus, Periacqueductal Grey Nucleus (Fig. 1c)

Micturition is similar to an on–off switching circuit which maintains a reciprocal relationship between the urinary bladder and the urethra outlet, thus allowing urine storage and voiding. Storage reflexes are activated during bladder filling and are organised primarily in the spinal cord, whereas voiding is mediated by reflex mechanisms in the brain (Fowler et al. 2008). The complexity of the neuroanatomical circuit also presupposes a hierarchic control by the cerebral cortex, brainstem and cerebellum and coordination between CNS, PNS and peripheral ganglia.

Physiologically, the urine storage phase is an involuntary process regulated by sympathetic thoracolumbar and somatic sacral spinal cord segments (Uemura 2015; De Lahunta et al. 2015; Samson and Reddy 1982; Sundin and Carlsson 1972; Tish and Geerling 2020). During filling, the detrusor muscle is relaxed (i.e. in a compliance state) and can contain a continuous amount of urine without increasing intravesical pressure (Hu et al. 2016). The continuous and gradual filling of the bladder is registered by A-delta mechanosensitive receptors. The sensory information travels along the axon of the pelvic nerve in dogs (Kuru and Iwanaga 1966) and the pelvic and hypogastric nerve in cats (De Groat et al. 1981). Through the spinobulbar tract, this sensory information reaches the lateral pons, also known as the pontine storage centre, pontine micturition centre (PMC), or M-region, now known as Berrington’s nucleus (Uemura 2015; Gjone 1966). In rats, tracing neuronal pathways using the pseudorabies virus revealed that the pontine micturition is close to the locus coeruleus (Nadelhaft and Vera 1996). Another brainstem micturition nucleus has been identified in rats, called the L-region nucleus. The L-region nucleus controls the Onuf’s nucleus too. This nucleus activates EUS enabling it to contract to prevent urine leakage (Holstege et al. 1986). In human medicine, to identify brainstem micturition nuclei (Georgiadis et al. 2006), PMC or Barrington’s nucleus or M-region nucleus have been replaced by the pelvic organ stimulating centre (POSC). Neurons in this group project to parasympathetic neurons eliciting micturition and parasympathetic neurons to the uterus, the distal colon and rectum. In contrast, the L-region, caudal and ventrolateral to the POSC, is known as the pelvic floor stimulating centre. When this centre is stimulated, it activates, in turn, the pelvic floor via Onuf’s nucleus, including the EUS and the bulbocavernosus and ischiocavernosus muscles (Holstege et al. 1979; Holstege et al. 1986; Holstege et al. 2003; Holstege 2016).

If the bladder is not sufficiently filled, brainstem micturition nuclei send efferent information along the reticulospinal tract to neuronal cells bodies of the hypogastric nerve, pelvic nerve and pudendal nerve to prevent urine leakage and guarantee continuing urine filling. This information results in the contraction of IUS and relaxation of the bladder innervated by adrenergic fibres of the hypogastric nerve, contraction of the EUS with the facilitation of somatic efferent cellular neuronal cell bodies (Onuf’s nuclei) that makes up the pudendal nerve. This prevents urine leakage, and cholinergic efferent neuronal cell bodies in the pelvic nerve are inhibited. All these mechanisms prevent leakage and perpetuating filling. When the stretch receptors (A-delta mechanoreceptors) register the stretching limit, which is18 mL/kg or 50 cmH2O in anaesthetised dogs, an action potential is initiated (De Groat and Wickens 2013). The information again reaches the sacral segments of the spinal cord through the GVA axon of the pelvic nerve (De Lahunta et al. 2015). The impulse then ascends the spinal cord along the spinothalamic pathways reaching the micturition centres in the brainstem (Oliver et al. 1969; De Lahunta et al. 2015). Experimental reports on cats have demonstrated that afferent input from the bladder (De Lahunta et al. 2015) ascends, through spinal interneurons in the lateral funiculus, to a relay station in the central periaqueductal grey nuclei (PAG) which, in turn, through the lateral PAG, provides excitatory input to the PMC (De Groat and Wickens 2013; Gjone 1966).

The presumed interconnection between the central and lateral PAG implies that signal processing occurs in the PAG, probably enabling higher centres to control the excitatory input to the PMC. The integration at this level generates a motor discharge descending along the spinal cord through the reticulospinal tract towards the neuronal polls involved in micturition. In summary, the brainstem centres are mainly PMC, under the PAG control (Fowler et al. 2008; De Groat and Wickens 2013) and the L-region nucleus, which regulates EUS activity. In turn, the PAG is regulated by a cortical network with the pre-frontal cortex (PFC) as the main final voluntary trigger. The descending information allows the inhibition of the adrenergic efferent neuronal cell bodies of the hypogastric nerve with the relaxation of the IUS, the facilitation of the cholinergic efferent neurological cell bodies of the pelvic nerve with the contraction of the detrusor muscle and the inhibition of the somatic efferent neuronal cell bodies (Onuf’s nuclei) of the pudendal nerve with the relaxation of the EUS (Oliver et al. 1969; De Lahunta et al. 2015). However, triggering the voiding reflex is under strict voluntary control, enabling voiding to be planned at an acceptable time and place.

#### Suprapontine integration: Cerebral Cortex, Hypothalamus and Cerebellum (Fig. 1 b, c)

It is also important to highlight that urine is used as a marker for territorial demarcation or sexual attraction (a female lets the males know that she is in oestrus by leaving a scent trace, or a male marks the territory). Micturition does thus not take place randomly or involuntarily but is part of a rather complicated behaviour directly related to the survival of the individual or species. This means that spinal cord micturition control must also be under the regulation of the emotional motor system (De Lahunta et al. 2015). The limbic system's involvement in eliciting bladder contraction has already been demonstrated in cats (Holstege et al. 1986). Some authors have explicitly reported projections from the hypothalamic preoptic area to the PMC (Ding et al. 1999). The hypothalamic preoptic area projects to the PMC, conferring a “safe signal” that enables voiding.

Young kittens and all puppies represent one exception to forebrain involvement in micturition in which the sacral cord is capable of producing a micturition reflex. This reflex needs to be elicited by the mother licking the perineum of young animals (Holstege 1987). This behaviour stops after approximately four weeks post-partum, after which the supraspinal centres play an essential role (Holstege 1987). On the other hand, during bladder filling, such higher brain centres, particularly the pre-frontal cortex (De Groat and Wickens 2013), can suppress the excitatory signal to the PMC and thus prevent voiding or incontinence. When voiding is consciously desired, they can allow the PMC to be excited. In cats, stimulation of forebrain structures, such as the anterior cingulate gyrus, preoptic area of the hypothalamus, amygdala, red nucleus of the strict terminalis and septal nuclei, elicits bladder contractions (de Groat et al. 1993; Holstege 2005). However, most of these regions send fibres to the brainstem (de Groat 2002) only the pre-optic area projects to PMC (De Groat 2006). The direct projection of the pre-optic area to the PMC is the tool the emotional motor system needs to control the PMC and thus determine the beginning of micturition (De Groat and Wickens 2013). Control of the cerebral cortex is demonstrated, for example, in territorial marking (voluntary initiation of urination) or house training (inhibition of urination) as learned behaviour.

In addition, the voiding reflex is also influenced by other subcortical areas, such as the cerebellum (Bastide and Herbaut 2020). On the other hand, both in human medicine and in experimental animals, the non-exclusivity of the cerebellum concerning somatic functions is well known (Doba and Reis 1972; Zheng et al. 1982; Bradley et al. 1987). In fact, in humans, accumulated experiences and clinical evidence have revealed the important role played by the cerebellum in emotional behaviour and non-somatic activities, such as visceral responses associated with internal and external environmental changes (Zhu et al. 2006; Reis and Golanov 1997; Bastide and Herbaut 2020). Several pathways support the hypothesis of the involvement of the cerebellum in the micturition reflex modulation, although not many reports have been published. In humans and animals, the anterior/rostral vermis and fastigial nucleus (FN) mainly contribute to micturition (Bastide and Herbaut 2020). A direct cerebellum connection with PAG has been described in cats, and bidirectional neural pathways to PMC have also been reported (Dietrichs 1983; Dietrichs and Haines 2002). Abundant efferents also characterise the FN to pontine and bulbar visceral centres and the reticular formation (Bastide and Herbaut 2020). Numerous afferents from the medullary/pontine reticular formation, locus coeruleus, primary motor cortex and cortical motor areas on the medial wall and the hypothalamus have been described (Zhang et al. 2016). Direct and indirect bidirectional connections between the cerebellum and hypothalamus have also been reported. (Zhu et al. 2006). The cerebellum receives afferent input from the detrusor muscle and EUS (through pelvic and pudendal nerves) (Bradley and Teague 1969b). The cerebellar afferent and efferent neural pathways enable the cerebellum to play a modulator and coordinator role in the somato-visceral responses.

From a functional point of view, cerebellum activation has been demonstrated in response to bladder distension, and both inhibitory and excitatory functions have been proposed (Bradley and Teague 1969a, b). In cats, the cerebellum has been suggested as playing an inhibitory role (Bradley and Teague 1969a, b, Martner 1975). In dogs, cerebellectomy induces bladder overactivity indicating a tonic inhibitory influence over the micturition reflex (Nishizawa et al. 1995).

#### Neuroplasticity

Another important aspect, rarely reviewed in animals, is the neuroplasticity of the nervous system in the developmental changes in the mechanism of micturition in prenatal and postnatal periods. Before the nervous system has matured in the fetus, urine is likely eliminated from the bladder by non-neural mechanisms (Fowler et al. 2008). In contrast, in newborn kittens, voiding depends on an exteroceptive somato-bladder reflex mechanism (De Groat 2002; Fowler et al. 2008), as previously described in this article. The exteroceptive reflex is located in the sacral spinal cord and has an afferent pathway in the pudendal nerve and an efferent pathway in the pelvic nerve (De Groat 2006). The exteroceptive perineal-to-bladder reflex is essential for survival: if the mother leaves the kitten/puppy alone, it will show urinary retention (De Groat et al. 1975; Thor et al. 1989; Araki and De Groat 1997). This reflex becomes weaker with the maturation of the nervous system (Thor et al. 1989). The adult type of voiding, previously described in detail in this article, only emerges several weeks after birth (Thor et al. 1989). During maturation, synaptic connections are reorganised into bladder reflex pathways leading to the down-regulation of primitive spinal mechanisms and the up-regulation of mature supraspinal mechanisms (Thor et al. 1989). How the organism passes from a primitive condition to a mature one is not well understood. However, the information passes through the remodelling of glutamatergic interneuronal pre-ganglionic neuron synapses in the sacral parasympathetic nucleus (Thor et al. 1989; Araki and De Groat 1997). This synaptic plasticity is associated with the down-regulation of primitive spinal micturition reflexes and the appearance of mature supraspinal voiding control (Thor et al. 1989; Araki and De Groat 1997). Some studies have demonstrated that SCI in adult animals and humans, which interrupts brain–spinal cord connections, causes the re-emergence of the neonatal perineal-to-bladder reflex (Thor et al. 1986).

## Conclusions

The functions of the LUT to store and periodically eliminate urine (micturition) are regulated by a complex system involving the autonomic, PNS and CNS, which work together to control and modulate the activity of smooth and striated muscles of the bladder and urethra. The micturition reflex is modulated by the integration between the CNS and PNS allowing voiding in an appropriate site and situation. The integration with the limbic system also regulates the emotional and hormonal state. Visceral activities (bladder and urethra) are also regulated by the cerebellum, generating integrated and coordinated somatic-visceral responses to adapt to changes in internal and external environments.

Consequently, in healthy animals, micturition depends not only on the neuro-physiological pathway functions but is also influenced by changes in age (neuroplasticity), experience and learned behaviours.

## Data Availability

Data sharing does not apply to this article as no datasets were generated or analysed during the current study.

## Abbreviations

CNS: Central nervous system
PNS: Peripheral nervous system
NB: Neurogenic bladder
LUTD: Lower urinary tract disease
QoL: Quality of life
UTIs: Urinary tract infections
IUS: Internal urethral sphincter
EUS: External urethral sphincter
GVE: General visceral efferent
GVA: General visceral afferent
GSE: General somatic efferent
SCI: Spinal cord injury
PMC: Pontine micturition centre
POSC: Pelvic organ stimulating centre
PFC: Pre-frontal cortex
FN: Fastigial nucleus
